# Organ removal of maize increases peanut canopy photosynthetic capacity, dry matter accumulation, and yield in maize/peanut intercropping

**DOI:** 10.3389/fpls.2023.1266969

**Published:** 2023-11-24

**Authors:** Zhu Liu, Zhenwu Nan, Songming Lin, Weiwei Meng, Liyong Xie, Haiqiu Yu, Zheng Zhang, Shubo Wan

**Affiliations:** ^1^ College of Agronomy, Shenyang Agricultural University, Shenyang, Liaoning, China; ^2^ Shandong Academy of Agricultural Sciences, Jinan, Shandong, China; ^3^ School of Life Sciences, Qilu Normal University, Jinan, Shandong, China

**Keywords:** intercropping, organ removal, canopy photosynthetic capacity, dry matter accumulation, yield

## Abstract

In maize/peanut intercropping systems, shade from maize is a major factor in peanut yield reduction. Reasonable redundant organ removal of maize plants could alleviate this problem and improve intercropped peanut yields. We studied the influences of organ removal of maize on peanut canopy photosynthetic capacity, dry matter accumulation and yield in maize/peanut intercropping systems in 2021 and 2022. Five organ-removal treatments were performed on maize plants to ameliorate the light environments in the peanut canopy. Treatments consisted of removal of the tassel only (T1), the tassel with top two leaves (T2), the tassel with top four leaves (T3), the tassel with top six leaves (T4), the leaves below the second leaf below the ear (T5), with no removal as control (T0). The results showed that organ-removal treatment (T4) significantly improved the photosynthetically active radiation (PAR, 49.5%) of intercropped peanut canopy. It improved dry matter accumulation by increasing the canopy photosynthetic capacity (canopy apparent photosynthetic rate (CAP), leaf area index (LAI), and specific leaf area (SLA)), ultimately contributing to peanut yield by increasing pod number per plant. Also, the above results were verified by structural equation modeling. The yield of intercropped peanut reached the highest value at T4. At the level of intercropping systems, the land equivalent ratio (LER) peaked at T2 (1.56, averaged over the two years), suggesting that peanut and maize can coexist more harmoniously under T2 treatment. The T2 treatment increased peanut yield by an average of 7.1% over two years and increased maize yield by 4.7% compared to the T0 treatment. The present study suggests that this may be an effective cultivation measure to mitigate intercropping shade stress in terms of adaptive changes in intercropped peanut under maize organ removal conditions, providing a theoretical basis for intercropped peanut yield increase.

## 1 Introduction

Peanut (*Arachis hypogaea* L.) is an essential oilseed crop broadly cultivated in tropical and subtropical (Cui et al., 2018). However, peanuts are usually grown and harvested using a continuous monoculture system, leading to increased disease incidence and reduced yield and quality (Chen et al., 2014), also known as replant disease (Liu et al., 2022b). To alleviate the consecutive monoculture problem, a promising strategy has been adopted to use intercropping to provide multiple ecosystem services. Maize/peanut intercropping can increase the system productivity by taking advantage of the edge effects and symbiotic nitrogen fixation ability of peanut (Wang et al., 2021b), which is widely practiced in semi-arid areas to increase economic and ecological benefits (Zhang et al., 2020a). However, in this intercropping system, shade stress from maize, resulting from plant height and shape differences, limits peanut growth.

Shade is a common abiotic stress during crop growth and development, especially in intercropping systems (Hussain et al., 2021). In intercropping systems, shading by the higher crops alters the light environment and field microclimate experienced by the lower crops (Carruthers et al., 2000; Liu et al., 2017b; Su et al., 2023). These changes trigger certain metabolic changes and an imbalance of resource assimilation and distribution, thus causing changes in the crops’ morphology and growth (Hussain et al., 2019). Morphological changes could include elongated internodes, increased plant height, and thinner leaves (Gong et al., 2015; Fan et al., 2018). Growth changes could include decreased biomass, declined number of flowers, and lower yield (Chen et al., 2020). Previous research has illustrated that soybean is sensitive to shade stress from adjacent plants, leading to the reduction of stem diameter, root biomass, and plant biomass, ultimately decreasing the seed yield of soybean (Yang et al., 2017). Additionally, shade stress can severely inhibit the main processes of photosynthesis by decreasing the production of ATPs in the photosystem II (PSII) reaction center by hindering the electron flow rate (Terashima et al., 2005; Valladares and Niinemets, 2008; Yao et al., 2017). Minimizing the shading effects of taller crops on the lower crops through reasonable agricultural practices is a crucial way to alleviate yield losses in intercropping systems (Raza et al., 2019d). However, most of these attempts have been made to change the spatial and temporal niche differentiation of intercrops by optimizing row ratio configurations and sowing dates, with only a few studies focusing on the effect of changing the structure of higher crops on mitigating shade stress in intercropping systems.

The crop canopy architecture could be altered by partially removing organs (Raza et al., 2019a; Liu et al., 2020). As a C4 crop, maize features efficient photosynthetic production and high yield potential (Edwards et al., 2001; Zhao et al., 2013). Further improvement of maize yield depends on improving source-sink balance (Shekoofa et al., 2013; Liang et al., 2023). For maize, leaves in the middle of the canopy provide more photosynthetic products to grain than other leaves (Liu et al., 2015). The upper layer of leaves usually shades leaves in the middle layer of maize, and this type of self-shading results in the reduction of light interception, the acceleration of leaf senescence, and thus limits the grain development (Marchiori et al., 2014; Cao et al., 2021). Therefore, moderately removing redundant organs is a useful agronomic practice to optimize the canopy architecture of maize, which can improve resource use efficiency and increase grain yield (Xue et al., 2017). Liu et al. (2015) observed that optimal leaf removal (uppermost two leaves) of maize plants around silking can decelerate leaf senescence, enhance canopy photosynthetic capacity, and increase dry matter accumulation. Xue et al. (2017) also observed that the removing all or half of the leaves above the three-ear-leaves at anthesis increased photosynthetically active radiation at the ear. Cao et al. (2021) reported that removing a quarter of the leaf length per plant increased grain yield by improving photosynthetic characteristics and dry matter accumulation. Moreover, Gao et al. (2020) reported that detasseling had a positive effect on grain yield. Nevertheless, excessive removal could detrimentally affect the photosynthetic performance and growth of the left leaves (Liu et al., 2015; Raza et al., 2019c).

In maize/peanut intercropping systems, moderate removal of redundant organs in maize might be a more effective agronomic attempt since it can ameliorate the light environment, alleviate shade stress, and enhance the yield of intercrops. However, few studies have concentrated on the effects of removing the redundant organs of maize on the photosynthesis of peanut leaves and grain yield of intercrops under maize/peanut intercropping systems. Therefore, a two-year field experiment was conducted to determine the response of peanut plants for canopy photosynthetic capacity, dry matter accumulation, and grain yield to different levels of shade stress from maize, manipulated by organ removal treatments, under maize/peanut intercropping systems. We hypothesize that in maize/peanut intercropping systems, optimal removal of the redundant organs of the maize plants could (i) enhance the canopy photosynthetic capacity, (ii) increase the dry matter accumulation, and (iii) improve the grain yields of intercrops in maize/peanut intercropping system. We verified this hypothesis by comparing five different organ removal treatments with no removal. The objectives of this study were: (1) to determine how different organ removal treatments influence the canopy photosynthetic capacity and dry matter accumulation of peanut in maize/peanut intercropping systems; (2) to investigate how the shift in shading intensity under different organ removal treatments influence peanut yield in maize/peanut intercropping systems; (3) to investigate the association between canopy photosynthetic capacity and yield in different organ removal treatments. The results can provide theoretical references for high-yield cultivation and maize breeding research in maize/peanut intercropping systems.

## 2 Article types

### 2.1 Experiment site

**Figure 1:**
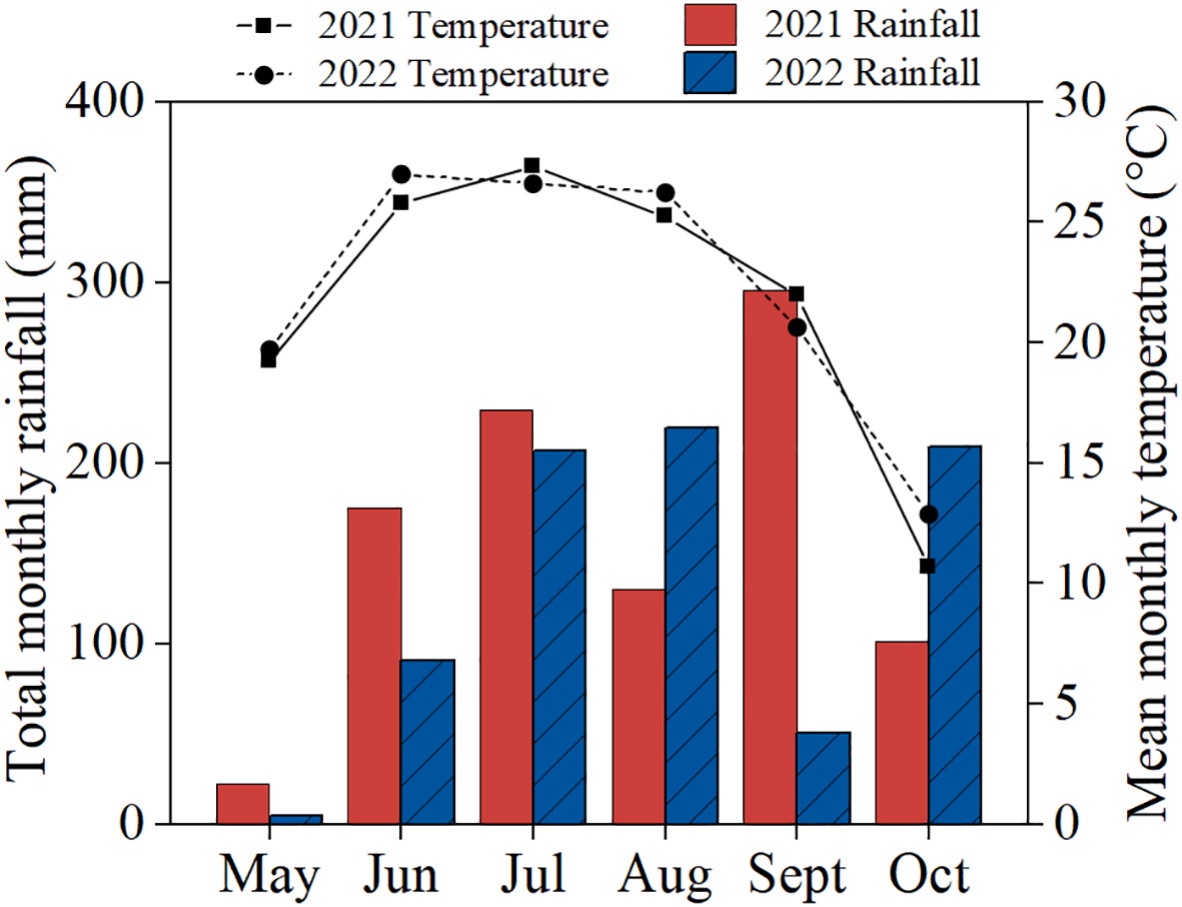
Monthly precipitation and mean air temperature of the research station during growing seasons in 2021 and 2022.

Field trial was conducted at Jiyang Experimental Station (116°58′E, 36°58′N) of Shandong Academy of Agricultural Sciences, Jinan City, Shandong Province, China. The area was characterized by a temperate continental monsoon climate with an annual average temperature and annual total rainfall of 14.9°C and 770 mm, respectively. The experiment site had a fluvo-aquic soil developed from the alluvial parent material of the Yellow River. The properties of the surface soil (0-20 cm layer) were the following: organic matter, 12.43 g kg^−1^; total N, 0.53 g kg^−1^; alkaline hydrolytic N, 54.74 mg kg^−1^; available P, 10.58 mg kg^−1^; available K, 96.45 mg kg^−1^. The weather data during the growing seasons are presented in **Figure 1**.

### 2.2 Experimental design

**Figure 2:**
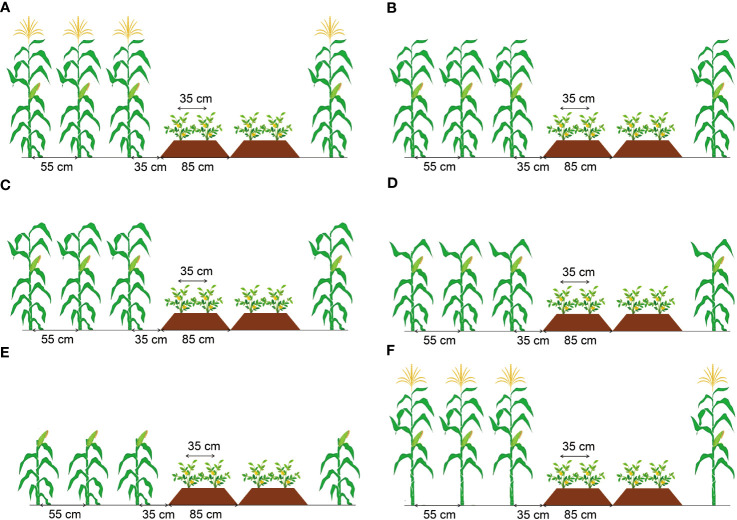
Schematic diagram of different organ removal treatments of maize in maize/peanut intercropping systems. T0 was the control with no removal **(A)**, T1 with the tassel removed **(B)**, T2 with the tassel and top two leaves removed **(C)**, T3 with the tassel and top four leaves removed **(D)**, T4 with the tassel and top six leaves removed **(E)**, T5 with the leaves below the second leaf below the ear removed **(F)**.

A randomized block design with six treatments (**Figure 2**) with three replications was used in this experiment. Treatments included: no removal (T0), remove the tassel (T1), remove the tassel and top two leaves (T2), remove the tassel and top four leaves (T3), remove the tassel and top six leaves (T4), and remove the leaves below the second leaf below the ear (T5). Maize plants were treated for organ removal when grown to seven days after silking (15^th^ August 2021 and 17^th^ August 2022).

Peanut (*Arachis hypogaea*, ‘Huayu-36’) was intercropped with maize (*Zea mays*, ‘Denghai-605’), and each strip in maize/peanut intercropping system contained four peanut rows and three maize rows (4:3) (**Figure 2**). Peanuts were planted on ridged beds with a width of 85 cm and a height of 15 cm. Two rows of peanuts were planted on each ridged bed with an inner row space of 35 cm and a spacing between plants of 14 cm. Maize was sown with a row spacing of 55 cm and a plant-to-plant spacing of 14 cm. The spacing between neighboring maize and peanut rows was 50 cm, and the total width of the strip was 350 cm.

Peanut and maize were sown on the second week of June and harvested on the first week of October in both years. Before sowing, 110 kg ha^−1^ of N (urea), 72 kg ha^−1^ P(calcium superphosphate), and 90 kg ha^−1^ K (potassium sulfate) were applied as base fertilizer. At the 11-leaf stage (V11) of the maize crop, an additional 60 kg ha^−1^ N was applied as a top dressing. The base fertilizer was based on the crop that requires less fertilizer, i.e., peanut. The amount of top dressing for maize was calculated based on the difference between the total fertilizer requirement and the base fertilizer. The field management, including weeds, pests, and diseases, was implemented according to the practices of local farmers.

### 2.3 Samples collection and measurement

#### 2.3.1 Photosynthetically active radiation

The photosynthetically active radiation (PAR) above the peanut canopy was measured using the AccuPAR LP-80 Ceptometer (Decagon Devices, Pullman, WA, USA) from 9:30 a.m. to 11:30 a.m. under clear skies. All measurements were taken at the peanut pod-setting (R3 (milk stage) for maize) and pod-filling stage (R5 (dent stage) for maize) and were repeated three times.

#### 2.3.2 Canopy apparent photosynthetic rate

Canopy apparent photosynthesis (CAP) was measured at the pod-setting stage and pod-filling stage of peanut with a LI-6400 portable gas-exchange photosynthesis system (LI-COR, Lincoln, NE, USA) (Zhang et al., 2020b). The assimilation box, which is 1 m long, 1 m wide, and 1 m high, was fitted with two fans, allowing over 95% of the solar radiation to pass through. The CAP measurements were conducted with negligible wind from 9:30 a.m. to 11:30 a.m. on a clear sunny day. The peanut system was enclosed in the assimilation box. Data were recorded when the CO_2_ concentration in the assimilation box decreased steadily. The gas exchange rate was measured three times for each treatment at 60 s intervals. The measured data was the net of total crop photosynthesis, crop respiration and soil respiration, called canopy apparent net photosynthesis (CAP’) (Zhang et al., 2020b). After measuring the CAP’, the above-ground portion of the plant in the assimilation box was cut down along the ground and removed. The assimilation box with an opaque black cloth was placed in its original position, and the measurements were repeated to detect soil respiration (R_soil_). CAP’ and R_soil_ were calculated according to Zhang et al. (2020b) as follows:


(1) $${\text{CAP' (or R}}_{\text{soil}})=\frac{(c_{0}-c_{1})\times V}{(t_{1}-t_{0})\times S}\times (\frac{44}{22.4}\times \frac{P}{101.3}\times \frac{273}{273+T})$$

where c_0_ and c_1_ are the initial and final concentrations of CO_2_ (mg L^−1^), t_0_ and t_1_ are the start and end times (s), V is the assimilation box volume (L), S is the ground area (m^2^), P is the air pressure (Pa), and T is the air temperature (°C).

The CAP was calculated as follows:


(2) $$\text{CAP}=CAP'-R_{soil}$$

#### 2.3.3 Leaf aera index and specific leaf aera

At the pod-setting stage (R3 for maize) and pod-filling stage (R5 for maize) of peanut, six representative peanut plants with uniform growth and vigor were selected from each plot. The leaf area was determined using the punching method (Cheng et al., 2023). Remove all leaves from the plant, randomly select 30 leaves and punch holes in the middle of the leaves using a puncher of known area. Record the number of punched leaves. The leaves were removed from the puncher, and the remaining leaves were dried separately until constant weight. The leaf area (LA) was calculated according to the formula:


(3) $$\text{LA=n}\times H\times \left(W_{p}+W_{r}\right)/W_{p}$$

where n is the number of punched leaves, H is the area of the hole, W_p_ is the weight of the leaves removed from the puncher, and W_r_ is the weight of the remaining leaves.

Then, the leaf area index (LAI) was determined by the total leaf area of the six peanut plants divided by the land area. The specific leaf area (SLA) was estimated by leaf dry weight divided by the LA.

#### 2.3.4 Dry matter sampling

Data on peanut were collected at the pod-setting stage (R3 for maize) and pod-filling stage (R5 for maize) in both years. At each sampling time, six representative peanut plants with the same growth and vigor were selected from each plot. These samples were divided into leaf, stem, root, and pod. All plant samples were placed in the oven at 105°C for 30 min and then at 75°C to constant dry weight.

#### 2.3.5 Yield and yield component

At maturity, pods were collected from the entire peanut strip in 2 m row length to determine peanut yield. Moreover, an entire maize strip in intercropping in 5 m row length in each plot was harvested to measure maize yield. For peanut, pod number per plant, 100-pod weight, 100-kernel weight, and shelling ratio were measured for all plants in the sampling area. Yields of intercropped peanut and intercropped maize in this article were based on the net area.

#### 2.3.6 Land equivalent ratio

The land equivalent ratio (LER) was calculated to measure the yield advantage of intercropping (Mead and Willey, 1980). The formula is as follows:


(4) $$\text{LER}=pLER_{p}+pLER_{m}=\frac{Y_{ip}}{Y_{mp}}+\frac{Y_{im}}{Y_{mm}}$$

where pLER_p_ and pLER_m_ are the partial land equivalence ratios of peanut and maize, respectively. Y_ip_ and Y_mp_ are the pod yields of peanut in intercropping and monoculture, respectively; Y_im_ and Y_mm_ are the grain yield of maize in intercropping and monoculture, respectively. The LER value >1 indicates that intercropping system has yield advantage.

### 2.4 Statistical analysis

The data was collected in Excel 2018. One-way analysis of variance (ANOVA) was executed using SPSS 26.0 (SPSS Inc., Chicago, IL, USA), and the least significant difference (LSD) was used to test the significance of differences at a 5% probability level. The graphs were drawn using Origin 2022 (OriginLab Crop., Northampton, MA, USA). Data were presented as the mean ± standard deviation based on repeated measurements. The AMOS 24 (SPSS Inc., Armonk, NY, USA) was used to build a structural equation model (SEM) to explore the effects of variables on changes in grain yield and the relationships among the variables.

## 3 Results

### 3.1 Photosynthetically active radiation

**Figure 3:**
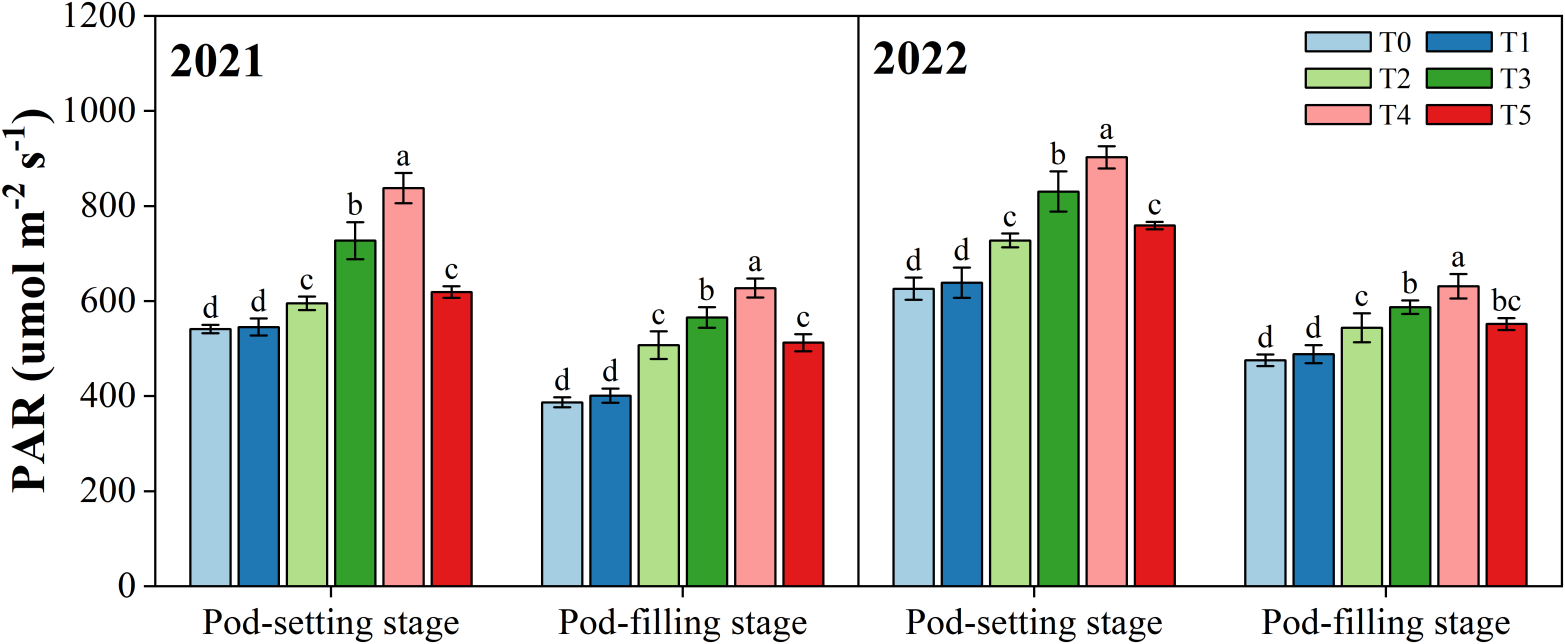
Effects of organ removal on canopy photosynthetically active radiation (PAR) of peanut in maize/peanut intercropping system in 2021 and 2022. Data are expressed as the mean of three replicates, and bars represent standard deviations (n = 3). Means do not share the same letters in the column differ significantly at *p*< 0.05.

The photosynthetically active radiation (PAR) of the peanut canopy was improved after the organ removal of maize in intercropping systems (**Figure 3**). At the pod-setting stage in the 2021 and 2022 growing seasons, PAR under T2, T3, T4, and T5 were significantly (*p*< 0.05) higher than that under T0, with an average increase of 13.2%, 33.6%, 49.5%, and 17.8%, respectively. However, the T1 treatment did not improve the light environment of peanut significantly because it only removed the tassel of maize. Similar results were observed at the pod-filling stage. T2, T3, T4, and T5 had a significant increase of 22.7%, 34.8%, 47.4%, and 24.2% (averaged over two years) in PAR of the peanut canopy relative to T0, respectively.

### 3.2 Canopy apparent photosynthesis

**Figure 4:**
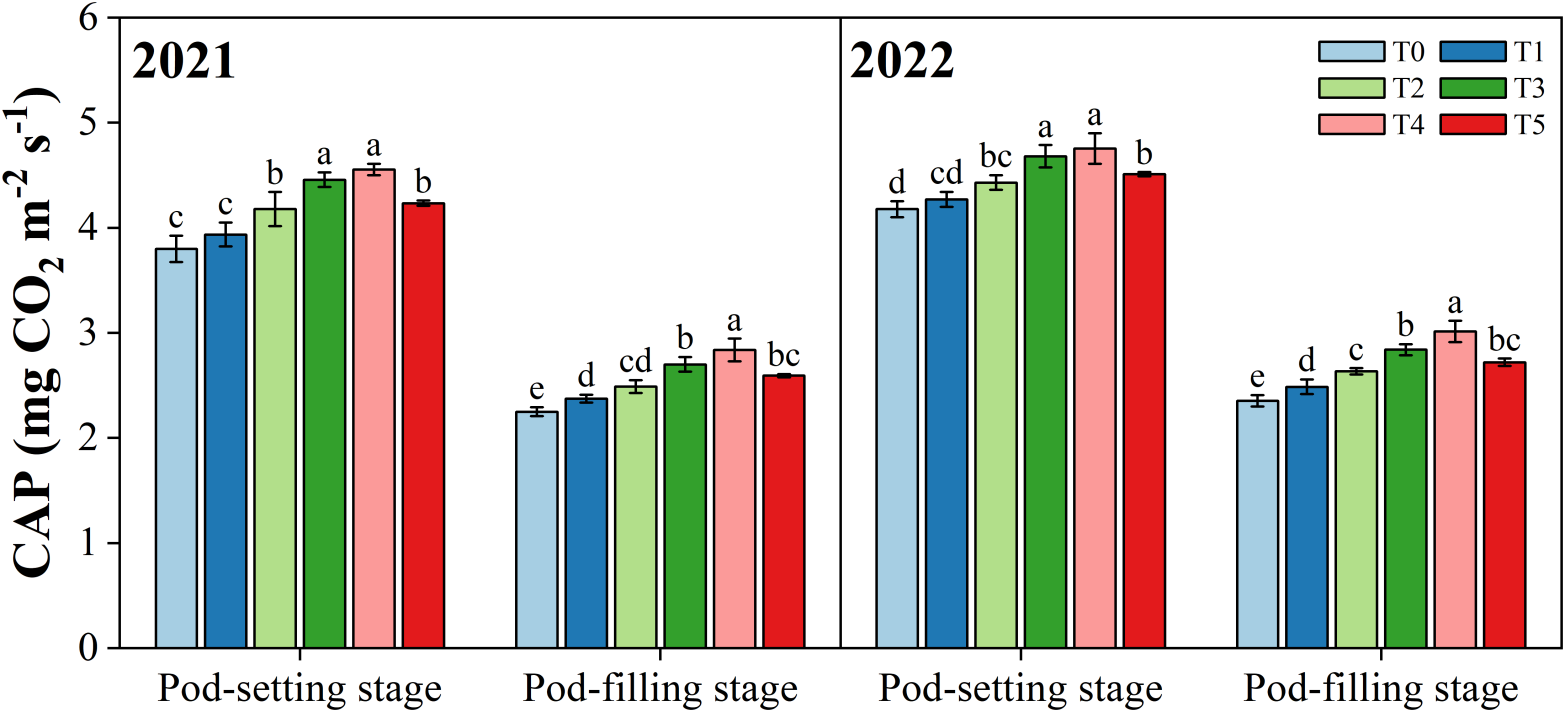
Effects of organ removal on canopy apparent photosynthesis (CAP) of peanut in maize/peanut intercropping system in 2021 and 2022. Data are expressed as the mean of three replicates, and bars represent standard deviations (n = 3). Means do not share the same letters in the column differ significantly at *p*< 0.05.

Different organ removal treatments significantly changed the canopy apparent photosynthesis (CAP) of peanut plants (*p*< 0.05) (**Figure 4**). Overall, in both years, the CAP value decreased from the pod-setting stage to the pod-filling stage and was maximized in the T4 treatment at the same growth stage. Compared with T0 treatment, T1, T2, T3, T4, and T5 treatments increased the CAP (mean of two years) by 2.9%, 8.0%, 14.7%, 16.7%, and 9.6% at the pod-setting stage, and 5.6%, 11.3%, 20.4%, 27.2%, and 15.4% at the pod-filling stage, averaged over two years, respectively.

### 3.3 Leaf area index

**Figure 5:**
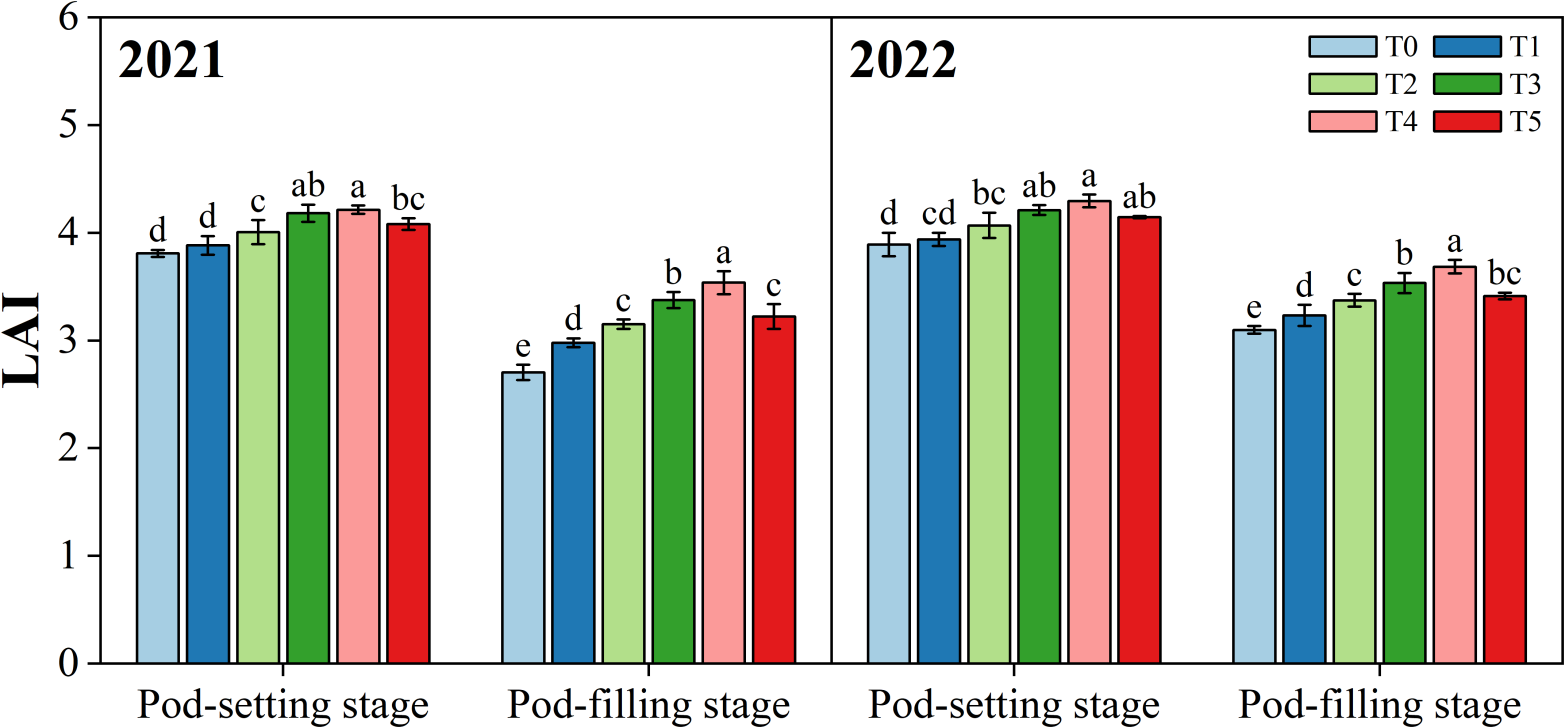
Effects of organ removal on leaf area index (LAI) of peanut in maize/peanut intercropping system in 2021 and 2022. Data are expressed as the mean of three replicates, and bars represent standard deviations (n = 3). Means do not share the same letters in the column differ significantly at *p*< 0.05.

Organ removal treatments had different effects on the leaf area index (LAI) of peanut plants at two stages (*p*< 0.05) (**Figure 5**). At the pod-setting stage, with the exception of the T1 treatment, the differences between the organ removal treatments (T2, T3, and T4) were non-significant but all significantly higher than T0. At the pod-filling stage, the highest LAI values were noticed under treatment T4, followed by T3, T5, T2, T1, and T0 in both years.

### 3.4 Specific leaf area

**Figure 6:**
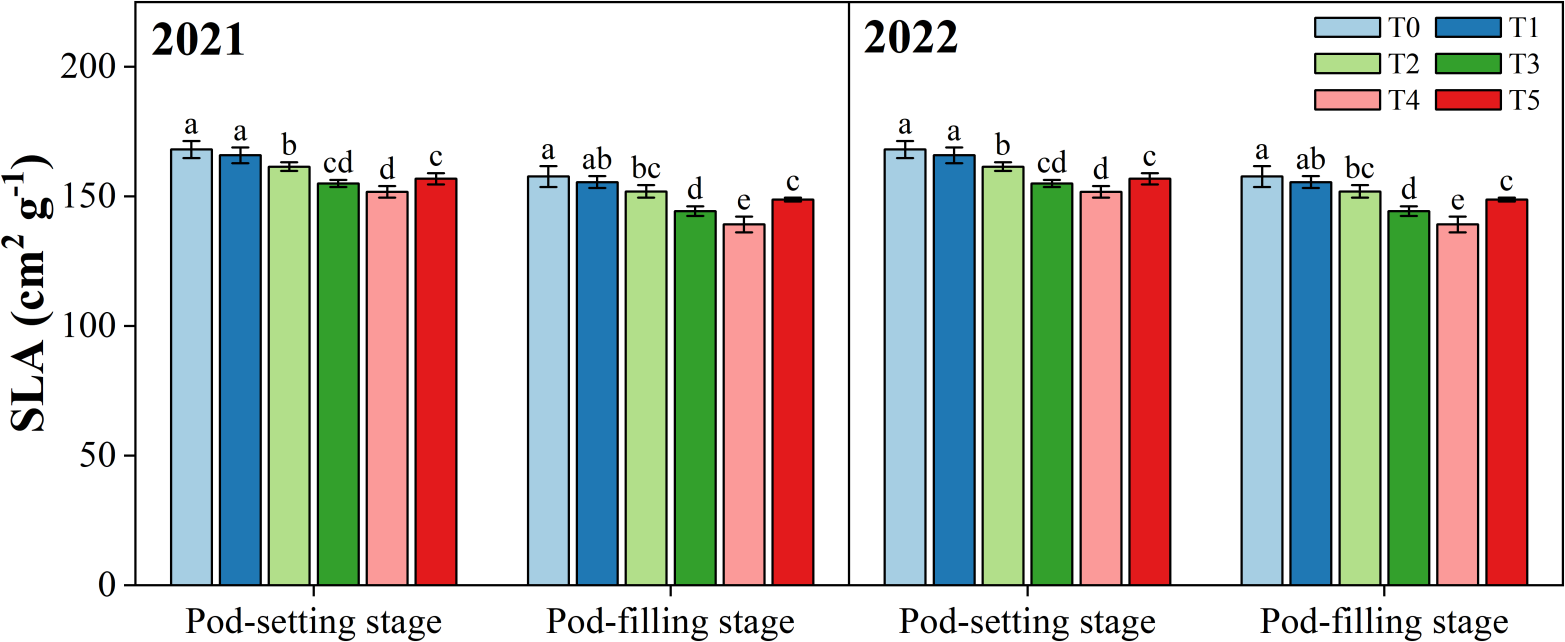
Effects of organ removal on specific leaf area (SLA) of peanut in maize/peanut intercropping system in 2021 and 2022. Data are expressed as the mean of three replicates, and bars represent standard deviations (n = 3). Means that do not share the same letters in the column differ significantly at *p*< 0.05.

**Figure 6** shows the effect of organ removal on the specific leaf area (SLA) of peanut at the pod-setting and pod-filling stages, respectively. SLA displayed a significantly decreasing trend in response to organ removal treatments compared to T0, except for T1, where the difference was non-significant. At the pod-setting stage, the minimum values for SLA were observed in T4 in 2021 (154.2 cm^2^ g^-1^) and 2022 (151.8 cm^2^ g^-1^), not significantly different from T3. At the pod-filling stage, T4 significantly decreased the SLA values by 11.5% in 2021 and 11.7% in 2022 compared with the T0.

### 3.5 Dry matter accumulation

**Figure 7:**
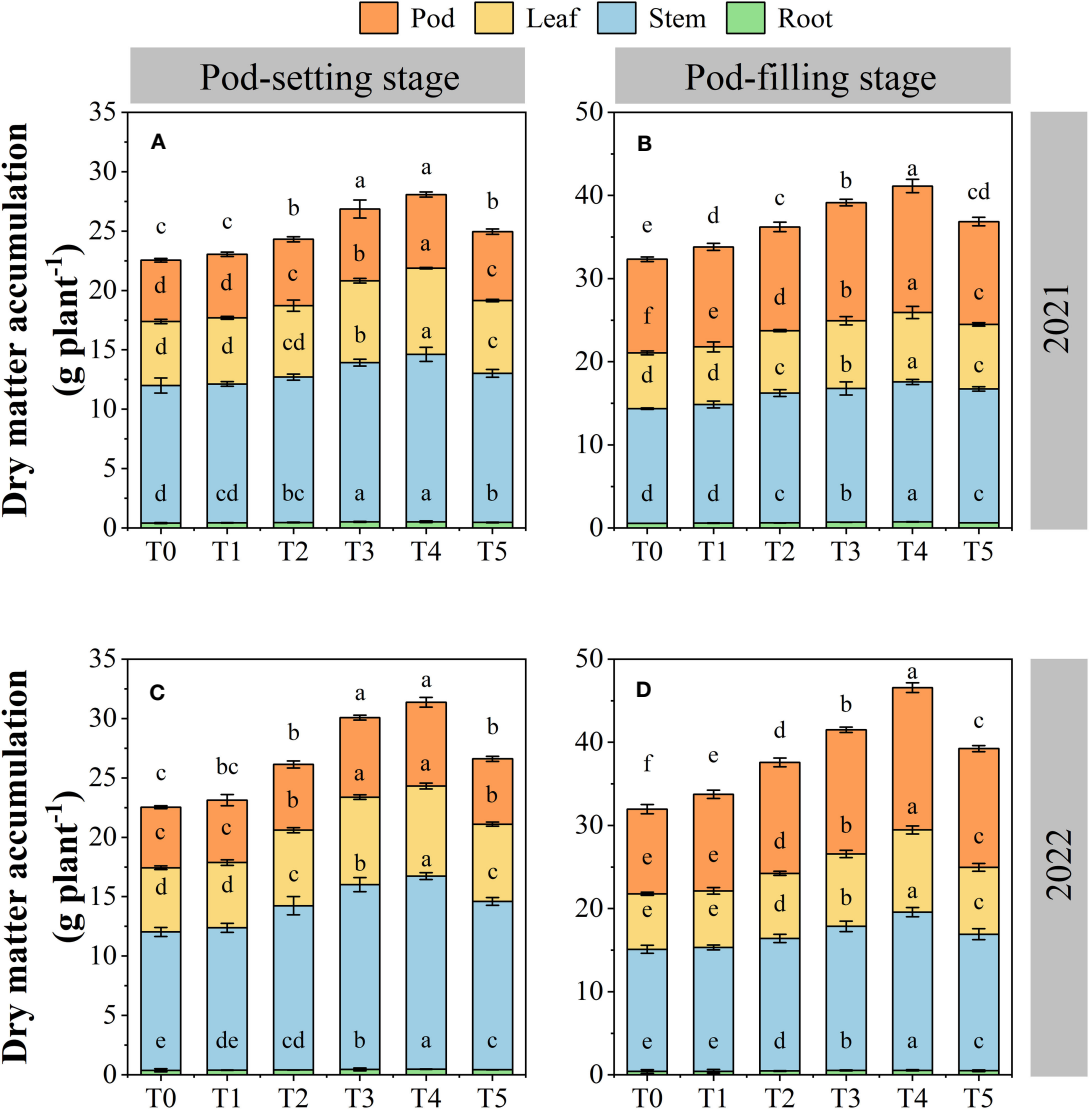
Effects of organ removal on dry matter accumulation of peanut in maize/peanut intercropping system in 2021 **(A, B)** and 2022 **(C, D)**. Data are expressed as the mean of three replicates, and bars represent standard deviations (n = 3). Means do not share the same letters in the column differ significantly at *p*< 0.05.

The dry matter accumulation was significantly affected by organ removal treatments (**Figure 7**). The dry matter accumulation increased in different organs and total plants as the growth period progressed. Across years and treatments, the order of dry matter accumulation of each organ at the pod-setting stage was stem > leaf > pod > root, and at the pod-filling stage was stem > pod > leaf > root, respectively. At the same growth stage, each peanut organ possessed a higher dry matter accumulation amount under organ removal treatments than the T0 treatment, and the T4 treatment showed the highest dry matter accumulation value. Across two years, the amount of dry matter accumulation at T4 treatment was increased by 25.7% for root, 30.6% for stem, 37.5% for leaf, and 28.2% for pod at the pod-setting stage, 32.0% for root, 29.4% for stem, 36.4% for leaf, and 42.1% for pod at the pod-filling stage, compared with T0 treatment, respectively.

### 3.6 Yield components, yield, and LER

The yield components of peanut in maize/peanut intercropping systems were significantly influenced by organ removal treatments (**Table 1**). In both years, organ removal treatments increased pod number per plant, 100-pod weight, 100-kernel weight, and the shelling ratio of peanut plants. On average over two years, the pod number per plant (12.83), 100-pod weight (173.43 g), 100-kernel weight (65.61 g), and shelling ratio (71.47%) achieved the highest value in T4. Compared with T0, T4 increased peanut pod number per plant by 28.0%, 100-pod weight by 12.5%, and 100-kernel weight by 6.8% in 2021, while 32.4%, 15.3%, and 5.9% in 2022, respectively. Averaging the two years of data revealed shelling rates ranging from 70.8% to 72.8%.

**Table 1:** Effects of organ removal on yield components of peanut in maize/peanut intercropping system in 2021 and 2022.

Year	Treatment	Pods per plant	100-pod weight (g)	100-kernel weight (g)	Shelling ratio (%)
2021	T0	11.07 c	163.40 d	63.20 d	70.79 b
	T1	11.50 c	164.96 d	63.91 cd	71.01 b
	T2	12.83 b	171.41 c	65.04 bc	71.25 b
	T3	13.43 ab	178.79 b	66.63 ab	71.98 a
	T4	14.17 a	183.88 a	67.48 a	72.14 a
	T5	12.77 b	172.76 c	65.66 b	71.46 ab
2022	T0	11.33 c	161.17 d	64.18 c	70.87 c
	T1	11.67 c	163.68 d	64.70 bc	71.25 c
	T2	12.83 bc	175.44 c	66.18 ab	71.68 bc
	T3	14.17 ab	181.43 ab	67.18 a	72.84 ab
	T4	15.00 a	185.87 a	67.96 a	73.43 a
	T5	13.67 ab	176.71 bc	66.33 ab	71.77 bc

The T0 refers to control (no removal); T1 refers to the removal of tassel; T2, T3, and T4 refer to the removal of tassel with two, four, and six leaves, respectively, from the top of a maize; T5 refers to the removal of the leaves below the second leaf below the ear. The SM and SP refer to the sole cropping system of maize and peanut, respectively. Means are averaged over three replicates. Means do not share the same letters in the column differ significantly at *p*< 0.05.

Intercropped peanut obtained the highest yield in the T4 treatment (**Table 2**). Compared with T0, treatment T4 increased the grain yield of peanut by 24.3%, averaged over the two years. The mean minimum peanut yield was recorded in T0. Although T4 significantly increased intercropped peanut yield, maize yield was 5.7% lower under the T4 treatment compared to T0. The grain yield of intercropped maize was significantly higher in T2 compared with other organ removal treatments (*p*< 0.05). On average, maize yield was increased by 4.7% under T2, compared with T0 treatment. Importantly, under T2 and T5 treatments, the yield of both peanut and maize was significantly higher than T0 (*p*< 0.05). Compared with T0, The T2 treatment increased peanut yield by 7.1% and maize yield by 4.7%, and the T5 treatment increased peanut yield by 7.0% and maize yield by 2.5%, averaged over two years. The yield of peanut showed the trend SP > T4 > T3 > T2 > T5 > T1 > T0, and that of maize exhibited the trend SM > T2 > T5 > T1 > T0 > T3 > T4.

**Table 2:** Effects of organ removal on grain yield and land equivalent ratio of maize and peanut in maize/peanut intercropping system in 2021 and 2022.

Years	Treatment	Yield (t ha^−1^)		pLER		LER
		Maize	Peanut	Maize	Peanut	
2021	T0	8.10 d	2.00 f	0.73 c	0.50 d	1.23 c
	T1	8.14 cd	2.04 ef	0.73 c	0.50 d	1.23 c
	T2	8.57 b	2.15 de	0.77 a	0.53 c	1.30 a
	T3	7.83 e	2.28 c	0.71 d	0.56 b	1.27 b
	T4	7.56 f	2.40 b	0.68 e	0.59 a	1.27 b
	T5	8.32 c	2.14 d	0.75 b	0.53 c	1.28 b
	SM	11.09 a				
	SP		4.05 a			
2022	T0	8.48 d	2.00 d	0.75 c	0.48 d	1.23 d
	T1	8.52 cd	2.02 d	0.75 c	0.48 d	1.24 d
	T2	8.78 b	2.15 c	0.78 a	0.52 c	1.29 a
	T3	8.29 e	2.23 c	0.73 d	0.54 b	1.27 c
	T4	8.08 f	2.37 b	0.71 e	0.57 a	1.28 ab
	T5	8.67 bc	2.13 c	0.77 b	0.51 c	1.28 bc
	SM	11.32 a				
	SP		4.16 a			

The T0 refers to control (no removal); T1 refers to the removal of tassel; T2, T3, and T4 refer to the removal of tassel with two, four, and six leaves, respectively, from top of a maize; T5 refers to the removal of the leaves below the second leaf below the ear. The SM and SP refer to sole cropping system of maize and peanut, respectively. Means are averaged over three replicates. The yields of intercropped crops were calculated according to the net area. Means do not share the same letters in the column differ significantly at *p*< 0.05.

In this study, the total land equivalent ratio (LER) values were greater than one in all organ removal treatments under the maize/peanut intercropping systems (**Table 2**). The values of pLER_m_ (partial land equivalent ratio of maize) were greater than the corresponding pLER_p_ (partial land equivalent ratio of peanut) values. However, the LER of peanut was considerably improved in T2, T3, T4, and T5 compared to T0 treatment, and T4 increased the pLER of peanut by 18.0% in 2021 and 18.8% in 2022 compared to T0. Overall, under maize/peanut intercropping systems, T2 treatment had the highest LER value, with an average of 1.30 for both years. T2 increased the pLER of peanut by 6.7% and maize by 4.6% compared to T0.

### 3.7 Correlation analysis

**Figure 8:**
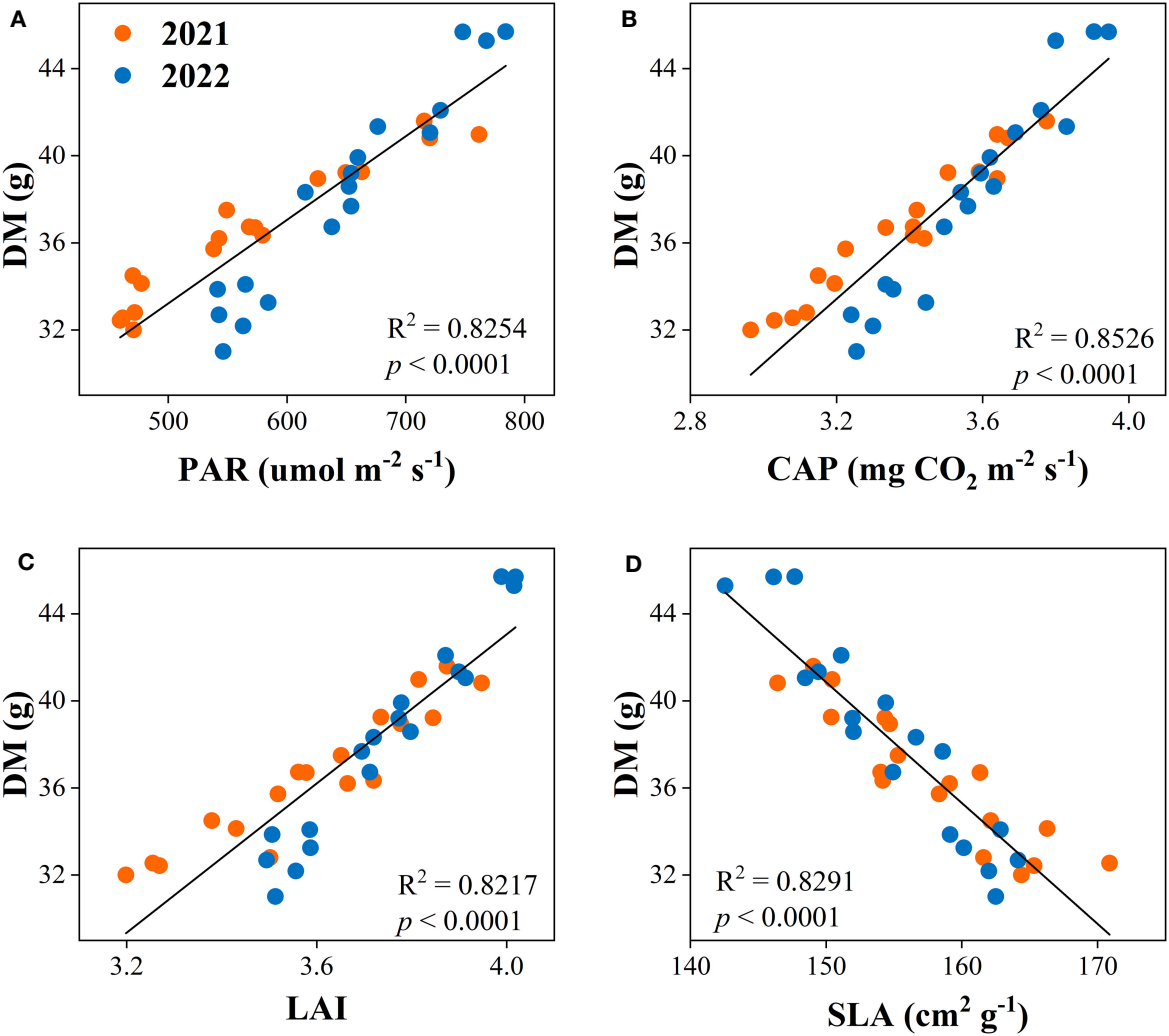
Relationships between dry matter accumulation (DM) and canopy photosynthetically active radiation (PAR, **A**), canopy apparent photosynthetic rate (CAP, **B**), leaf area index (LAI, **C**), and specific leaf area (SLA, **D**) of the peanut crop.

**Figure 9:**
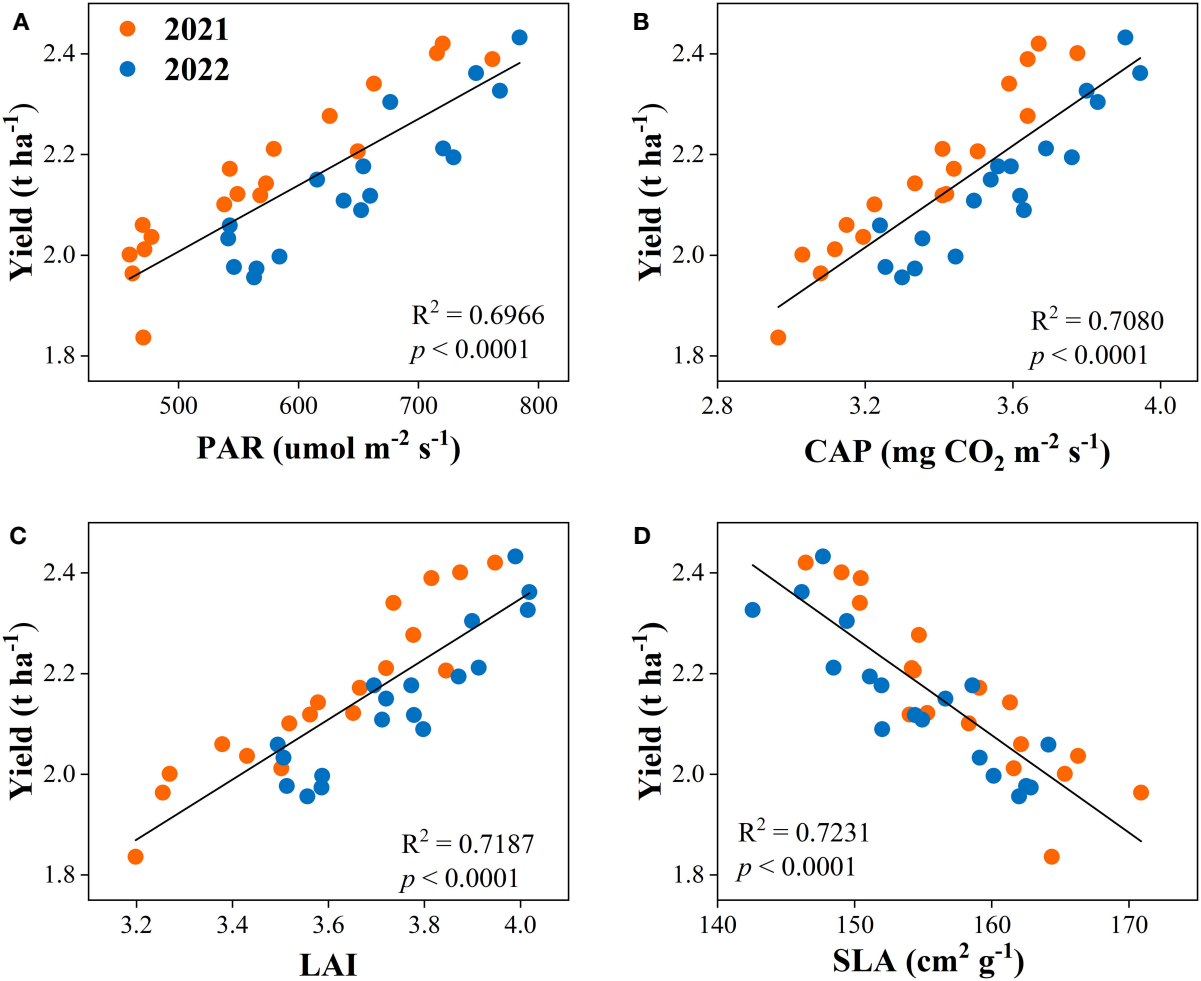
Relationships between yield and canopy photosynthetically active radiation (PAR, **A**), canopy apparent photosynthetic rate (CAP, **B**), leaf area index (LAI, **C**), and specific leaf area (SLA, **D**) of the peanut crop.

The correlation analysis was used to determine the relationship between dry matter accumulation and yield with canopy apparent capacity (PAR, CAP, LAI, and SLA). The result showed that the dry matter accumulation had positive relationships with PAR, CAP, and LAI and was negatively related to SLA (**Figure 8**). Equally, yield was positively associated with PAR, CAP, and LAI, while negatively correlated with SLA (**Figure 9**).

**Figure 10:**
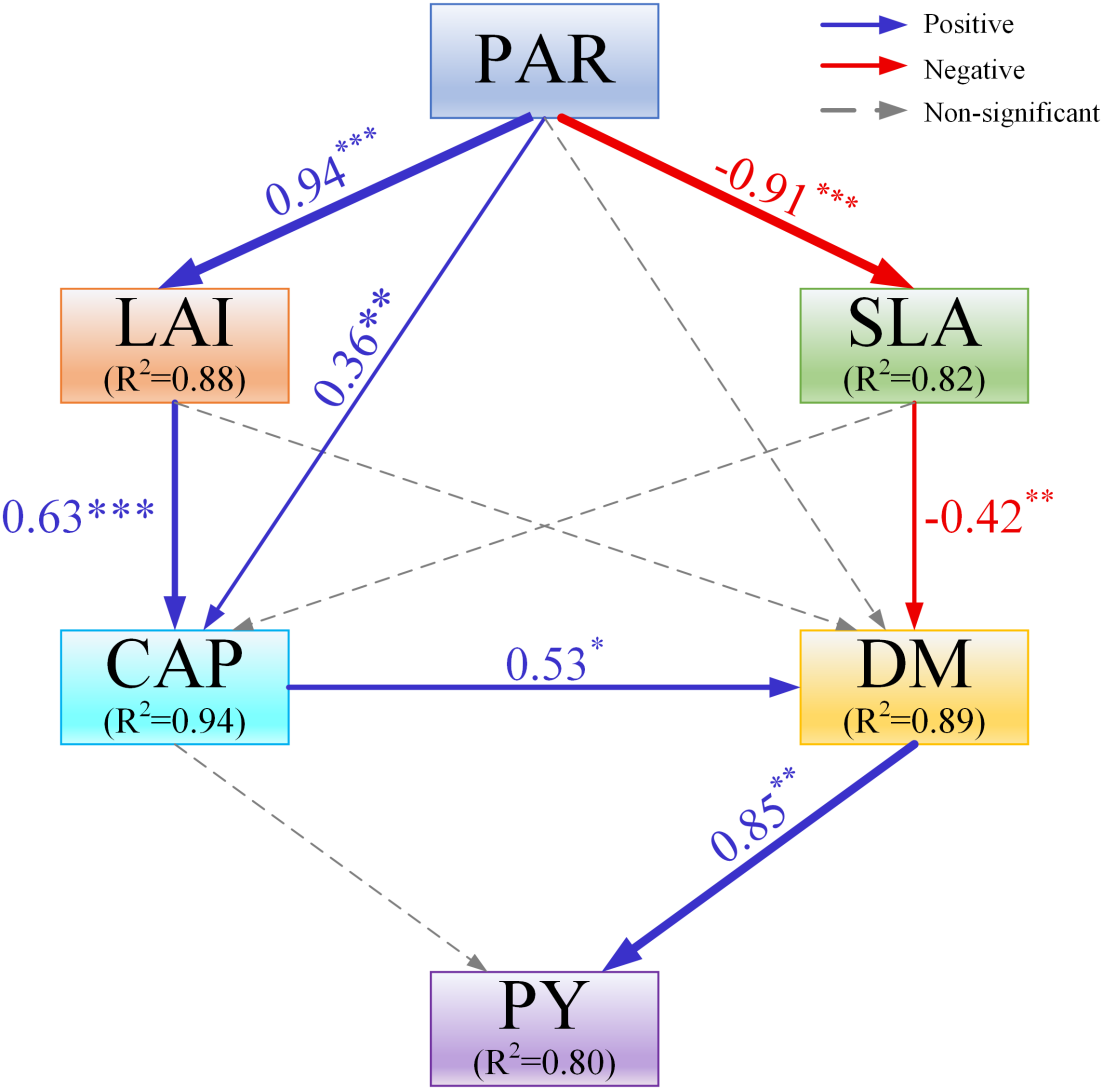
The structural equation modeling (SEM) assessing the effects of canopy photosynthetically active radiation (PAR), canopy apparent photosynthetic rate (CAP), leaf area index (LAI), specific leaf area (SLA), and dry matter accumulation (DM) on the response of peanut yield (PY). Blue, red, and grey arrows indicate positive, negative and nonsignificant relationships, respectively, with the thickness representing the extent of influence. Numbers beside arrows denote standardized path coefficients. *, *p*< 0.05; **, *p*< 0.01, ***, *p*< 0.001.

The structural equation modeling (SEM) was constructed to explain the direct and indirect relationships (**Figure 10**). The results showed that PAR had a direct effect on LAI, SLA, and CAP. Dry matter accumulation (DM) was the important variable that had a direct and positive effect on the changes in peanut yield (PY). PAR, LAI, SLA, and CAP all indirectly affected the changes in PY through the DM.

## 4 Discussion

### 4.1 Organ removal changes the light environments and canopy photosynthetic capacity of peanut canopy

Solar radiation, especially photosynthetically active radiation (PAR), plays a crucial role in plant photosynthetic processes, directly affecting growth, development, and yield (Wang et al., 2015; Yao et al., 2016). Previous studies have demonstrated that crop architecture and intercropping configuration can change PAR distribution and have an impact on the morphology and growth of plants (Gong et al., 2020). Similarly, in this study, different degrees of maize organ removal enhanced the canopy PAR of the peanut in maize/peanut intercropping systems (**Figure 3**), showing that the light environment of peanuts has been improved. Specifically, the highest PAR value was found at T4 treatment (removal of the tassel with top six leaves), which was remarkably higher than T0. The main reason for increased PAR was a reduction in shade stress due to maize organ removal. Similar results were observed by Raza et al. (2019b) in maize/soybean intercropping systems. Moreover, redundant organ removal was shown to affect the light transmittance of maize leaves in the middle layer positively (Xue et al., 2016; Xue et al., 2017). Thus, changing the maize architecture can improve the light environment at the peanut canopy under maize/peanut intercropping systems.

Canopy photosynthetic capacity refers to the photosynthetic capacity of crops at the population level (Bondada and Oosterhuis, 2001; Song et al., 2023). It is strongly correlated with leaf physiological and morphological characteristics such as canopy apparent photosynthesis (CAP), leaf area index (LAI), and specific leaf area (SLA) (An and Shangguan, 2008; Wang et al., 2021c). Improving canopy photosynthetic capacity at critical growth stages is essential to increase crop production (Wells et al., 1982; Yang et al., 2019).

The photosynthetic activity for different leaf morphologies and canopy structures can be reflected by CAP, which is affected by shifts in the light environment (Zelitch, 1982; Kim et al., 2006; Gong et al., 2020). However, previous studies have mainly focused on the single-leaf photosynthetic capacity, and relatively little is known about the population photosynthetic capacity. This study investigated the impact of different organ removal treatments on the CAP of peanuts in maize/peanut intercropping systems. Compared with T0, all organ removal treatments considerably increased the CAP of peanut, and the CAP achieved the highest value in T4 (removal of the tassel with top six leaves) (**Figure 4**). The higher CAP values for peanut were attributed to the enhancement of the light environment through removing organs, thus reducing the light competition (Zou et al., 2019). Similar to our findings, higher photosynthetic characteristics were reported under improved light conditions in different intercropping systems (Raza et al., 2019b; Huang et al., 2022). These findings have revealed that crop leaves can alter their photosynthetic properties to accommodate changing light environments (Huang et al., 2011; Wang et al., 2021a; Su et al., 2023). Additionally, the SEM results showed that LAI had a direct and positive effect on CAP (**Figure 10**), which partly explain the significant increase in CAP, suggesting that CAP was tightly associated with variation in leaf area (**Figure 5**) (Liu et al., 2015; He et al., 2023).

The leaf is the major photosynthetic organ, and leaf area has a direct effect on the amount of PAR intercepted by plants (Zhang et al., 2020a). LAI is an essential indicator of canopy structure performance, and the improvement in LAI can increase light interception and radiation utilization efficiency (Vaesen et al., 2001; Liu et al., 2017c). Numerous studies have shown that, in intercropping systems, shading from higher crops reduces the LAI of lower crops (Su et al., 2014; Cheng et al., 2022). For instance, a previous study has reported that shade from maize severely limited the development of leaf area and reduced the expansion and proliferation of leaves in soybean plants under maize/soybean intercropping systems (Wu et al., 2017a). In the present study, LAI of peanut under removal treatments increased by 1.4% to 15.9% compared with no-removal treatment (T0) (**Figure 5**), suggesting that maize organ removal can alleviate shade stress and promote leaf growth and development. The SEM results demonstrated that PAR had positive effects on LAI (**Figure 10**), indicating increased PAR in peanut canopy due to the removal of redundant organs from maize can increase LAI in peanut plants. These results are in line with findings on maize/soybean intercropping systems (Raza et al., 2019a; Raza et al., 2019b).

SLA is a vital parameter reflecting light interception and light utilization efficiency, which usually affects photosynthetic capacity (Dijkstrai and Lambers, 1989; de Avila Silva et al., 2021). Changes in SLA derive from shifts in leaf thickness and density (Witkowski and Lamont, 1991; Evans and Poorter, 2001). Previous studies demonstrated that plants grown in high light generally have thick leaves with a low SLA while increasing their SLA to optimize light capture and utilization when exposed to shade conditions (Evans and Poorter, 2001; Feng and van Kleunen, 2014). Similarly, lower crops in intercropping systems were found to have relatively high SLA (Liu et al., 2017b; Wu et al., 2017b). That is because, shaded by higher crops, higher SLA can provide more leaf area for light interception (Gutschick and Wiegel, 1988; Feng and Fu, 2008; Zhou et al., 2023). In our field experiment, SLA values of intercropped peanut were lower in five organ removal treatments than in the control treatment with no removal (T0) (**Figure 6**). This result is mainly due to organ removal from the maize plant reducing shade stress and improving the light environment of the peanut canopy, thereby increasing the light-harvesting efficiency of the peanut leaves (Raza et al., 2019a). Low SLA values generally imply that leaves have a high photosynthetic capacity, which partially explains the increase in CAP under the organ removal treatment (Niinemets and Sack, 2006; Feng et al., 2008; Perthame et al., 2022).

### 4.2 Dry matter accumulation and yield formation of intercropping composite population

Dry matter production and accumulation are key to crop yield formation. Increased canopy photosynthetic capacity of leaves is the main factor affecting the dry matter yield of peanut. Previous studies confirmed that decreased PAR transmission rate significantly reduced dry matter accumulation in soybean plants under maize/soybean intercropping (Yang et al., 2014). In this study, we found that through organ removal of maize in maize/peanut intercropping systems, peanut can harvest and utilize enough sunlight to complete biochemical and physiological processes, maintaining a high dry matter accumulation. Organ removal treatments increased the dry matter accumulation of peanut at pod-setting and pod-filling stages, and the T4 treatment obtained the highest values. Organ removal increased the photosynthetic properties of the peanut canopy, thereby enhancing the nutrient uptake in crops, which may account for the increase in dry matter accumulation (Baligar et al., 2006; Gao et al., 2023). Previous studies on proso millet/mung bean (Gong et al., 2020), maize/soybean (Raza et al., 2019a), and waxy sorghum/soybean (Wang et al., 2021a) intercropping systems reported similar results. Additionally, we have further assessed the response to organ removal treatments of dry matter distribution in peanut root, stem, leaf, and pod, finding that T4 treatment increased the distribution of dry matter accumulation in pod at the pod-filling stage. These results indicate that organ removal of maize can increase the total dry matter accumulation and facilitate dry matter transport from nutrient organs to reproductive organ in peanut in maize/peanut intercropping systems. It was also the direct reason for the increase of peanut yield (**Figure 10**).

In intercropping systems, lower crops are often shaded by higher crops, leading to reduced yields in lower crops (Zou et al., 2023). Our findings agree with the common observation in intercropping systems that maize was the dominant crop species in maize/peanut intercropping systems. This study showed that organ removal of maize had a significant effect on the yields of maize and peanut in intercropping systems. Yield of peanut was increased under different organ removal treatments especially in T4 treatment. According to the SEM results, the peanut yield is mainly determined by DM (**Figure 10**). PAR, LAI, SLA, and CAP all indirectly affected the changes in peanut yield through the DM. Therefore, we may propose that an optimum maize organ removal treatment improves the peanut canopy PAR by altering the maize canopy architecture, thereby increasing canopy photosynthetic capacity and dry matter accumulation, thus obviously increasing peanut yield. Moreover, the increase in peanut yield was attributed to an increment in pod number per plant (**Table 1**). The increase in maize yield of top organ removal treatments (T2) may be largely due to ameliorated light distribution, enhanced photosynthetic capacity, and altered source-sink ratio (Liu et al., 2020). Previous studies have shown that the PAR was intercepted by leaves above 2/3 of the height of the maize canopy after the tasseling stage (Tian et al., 2022). The contribution of lower strata leaves to yield formation is much lower than their potential (Liu et al., 2020). Removing partial lower leaves of maize can reduce the ineffective consumption of limited resources and increase resource utilization efficiency, which is also responsible for the increased maize yield in the T5 treatment (Liu et al., 2022a). However, excessive removal treatments (T3 and T4) reduced the maize yield. The reason may be the loss of too many leaves leading to a decrease in the LAI and photosynthetic rate of maize (Raza et al., 2020). Similar results were reported in past studies (Liu et al., 2017a; Raza et al., 2019a).

Intercropping advantage can be quantified by the land equivalent ratio (LER) (Li et al., 2020; Liu et al., 2020; Liu et al., 2023). In this study, the LER values of all treatments were greater than one in maize/peanut intercropping systems, revealing that maize/peanut intercropping systems have higher land resource utilization efficiency than monocultures, which is a promising way for farmers with limited land resources (Wang et al., 2023). Specifically, the LER of the intercropping system achieved the highest value of 1.30 at T2 treatment (removal of the tassel with top two leaves) over two years (**Table 2**), which means that 30% more farmlands would be needed for peanut and maize in monoculture to equal the yield of maize and peanut intercropping (Raza et al., 2019e). Therefore, T2 is the optimum organ removal level in maize/peanut intercropping, which substantially increased the peanut yield (by 7.1%) and also significantly increased the maize yield (by 4.7%) compared to T0 treatment. These results demonstrate that maize/peanut intercropping is a high land-use system under optimum organ removal treatments.

## 5 Conclusion

The results of this study indicated that the level of organ removal positively affected the canopy photosynthetic capacity and dry matter accumulation of peanut in maize/peanut intercropping systems. Organ removal treatments enhanced the PAR on peanut canopy and improved the light environment, which increased the CAP and LAI of peanut plants, thus promoting dry matter accumulation. Moreover, organ removal alleviated shade stress in intercropping, as evidenced by the reduced SLA of peanut plants. Peanut and maize obtained greater yields in T1, T2, and T5 treatments under the maize/peanut intercropping system. However, heavy removal (T3 and T4 treatments) increased peanut yield but resulted in a significant decrease in maize grain yield. Overall, optimal organ removal (removal of the tassel and top two leaves of maize) of maize greatly improved the LER (1.30) of the maize/peanut intercropping system.

## Data availability statement

The raw data supporting the conclusions of this article will be made available by the authors, without undue reservation.

## Author contributions

ZL: Investigation, Visualization, Writing – original draft. ZN: Investigation, Writing – original draft. SL: Software, Writing – original draft. WM: Conceptualization, Writing – review and editing. LX: Writing – review and editing. HY: Writing – review and editing. ZZ: Writing – review and editing. SW: Supervision, Writing – review and editing.
